# A Rare Guillain-Barré Syndrome Variant with Multi-Ganglioside Reactivity: A Case of Severe Cranial Nerve Involvement

**DOI:** 10.31083/RN37744

**Published:** 2025-02-12

**Authors:** Laura Gómez-Dabó, Arnau Llaurado, Daniel Sánchez-Tejerina, Victoria González, Carmen Montalvo-Olmedo, Carlos Lázaro-Hernández, Marc Rodrigo-Gisbert, Samuel López-Maza, Maider Iza-Achutegui, Lídia Giramé-Rizzo, Nuria Raguer, Raúl Juntas

**Affiliations:** ^1^Department of Neurology, University Hospital Vall d’Hebron, Universitat Autònoma de Barcelona, 08035 Barcelona, Spain; ^2^Neuromuscular Diseases Unit, Neurology Department, University Hospital Vall d’Hebron, 08035 Barcelona, Spain; ^3^Department of Clinical Neurophysiology, Vall d’Hebron University Hospital, 08035 Barcelona, Spain

**Keywords:** anti-ganglioside antibody, disialosyl, Guillain–Barre syndrome, multiple cranial nerve, NeuNAc(α2-3)Gal, neuropathy, anticuerpo anti-gangliósido, disialosilo, síndrome de Guillain-Barré, multineuritis, NeuNAc(α2-3)Gal, neuropatía

## Abstract

**Introduction:**

We present a rare case of acute immune-mediated polyradiculoneuritis, a Guillain-Barré Syndrome (GBS) variant, manifesting as ophthalmoparesis-ataxia, facial diplegia, and acute bulbar palsy, accompanied by a unique autoimmune profile.

**Clinical Case:**

A 75-year-old female developed rapidly progressive symptoms, including bilateral non-reactive mydriasis, ptosis, complete ophthalmoplegia, bilateral facial weakness, tongue immobility, palatal paralysis, limb dysmetria, ataxia, and brisk generalized tendon reflexes, all while maintaining a preserved mental state. Symptoms emerged 10 days after a probable gastrointestinal infection. Severe bulbar dysfunction necessitated orotracheal intubation and a tracheotomy. Extensive cranial nerve involvement initially suggested a brainstem lesion, with oculomotor and acute bulbar palsy as prominent signs. However, brainstem and spinal magnetic resonance imaging along with cerebrospinal fluid analysis yielded negative results. Electromyography reveled a sensorimotor demyelinating polyradiculoneuropathy, and serum testing identified IgG antibodies targeting multiple gangliosides, including the disialosyl group and terminal NeuNAc(α2-3)Gal. Treatment with intravenous immunoglobulin (IVIG) led to gradual clinical improvement.

**Conclusions:**

This case highlights a rare and severe GBS phenotype characterized by reactivity to multiple gangliosides. It highlights the role of shared ganglioside epitopes in antibody-mediated neurological damage and expands the clinical spectrum of GBS variants.

## Abstract (es)

**Introducción:**

Presentamos un caso clínico de polirradiculoneuritis aguda inmunomediada que inicialmente se manifestó con oftalmoparesia-ataxia, diplegia facial y parálisis bulbar aguda, acompañada de un perfil autoinmune característico.

**Caso Clínico:**

Describimos el caso de una mujer de 75 años con clínica de progresión rápida incluyendo midriasis bilateral no reactiva, ptosis, oftalmoplejía completa, paresia facial bilateral, paresia lingual, parálisis del paladar, dismetría en todas las extremidades, ataxia y reflejos osteotendinosos aumentados de forma generalizada, con nivel de conciencia preservado. La clínica inició después de una posible infección gastrointestinal de aparición diez días antes. Su estado clínico empeoró rápidamente, requiriendo intubación orotraqueal y traqueotomía debido a un compromiso bulbar severo. La afectación concomitante de múltiples nervios craneales sugirió una lesión en el tronco encefálico, destacándose la parálisis oculomotora y bulbar aguda. La resonancia magnética del tronco encefálico y médula espinal, junto con las pruebas de líquido cefalorraquídeo, no mostraron alteraciones; la electromiografía objetivó una polirradiculoneuropatía desmielinizante sensitivo-motora. La prueba de anticuerpos antigangliósidos mostró positividad contra múltiples anticuerpos dirigidos al grupo dialosilo y al terminal NeuNAc(α2-3)Gal. El tratamiento con inmunoglobulinas se asoció a una mejoría gradual.

**Conclusiones:**

Nuestro caso ilustra la reactividad a múltiples gangliósidos, destacando los epítopos compartidos entre estas moléculas y la capacidad de un único anticuerpo para dirigirse a diversos tipos de gangliósidos, subrayando además un fenotipo extremadamente raro del síndrome de Guillain-Barré.

## 1. Introduction

Guillain-Barré Syndrome (GBS) is a clinically heterogeneous disorder characterized by a classic presentation of progressive ascending limb weakness with reduced or absent reflexes. While the current diagnostic criteria [1, 2] are widely employed in diagnosing most GBS patients, a noteworthy proportion fails to meet these criteria. In such instances, comprehensive neurophysiological studies and antiganglioside antibody testing can prove highly beneficial.

In this case report, we present the case of a patient with acute immune-mediated polyradiculoneuritis presenting initially with ophthalmoparesis-ataxia, facial diplegia, and acute bulbar palsy, accompanied by a characteristic autoimmune profile. Adherence to the CARE guidelines ensures that this case report meets the highest standards for clarity and completeness. The checklist is provided in the supplementary materials for reference (**Supplementary Material-CARE-checklist-English**).

## 2. Case Report

A 75-year-old female with hypertension, dyslipidemia, and asthma was admitted to the Internal Medicine Department because of a gastrointestinal illness. No specific pathogen identified, but empiric treatment with amoxicillin-clavulanic was initiated, resulting in clinical and fever remission within five days. However, two days after, she experienced rapidly progressive impairment of the bilateral III, IV, VI, VII, IX, X and XII cranial nerves. This manifested as bilateral ophthalmoplegia with complete ptosis, non-reactive mydriasis, facial palsy, tongue palsy, absence of palate elevation, abolished pharyngeal reflex, and flaccid dysarthria. The examination also revealed brisk generalized tendon reflexes, and bilateral upper and lower limb dysmetria. Motor and sensory system, and mental state examinations were normal. Within hours, the patient deteriorated with respiratory insufficiency, requiring endotracheal intubation and Intensive Care Unit admission.

Urgent cerebral and spinal magnetic resonance imaging (MRI) revealed mild non-specific cerebral chronic white matter changes, with normal brainstem and spinal parenchyma, absence of gadolinium enhancement, and permeability of blood vessels in magnetic resonance angiography. Lumbar puncture with cerebrospinal fluid (CSF) cytology and biochemical studies showed no abnormalities (0 cells/uL, glucose 86 mg/dL, protein 38 mg/dL). CSF extensive microbiologic testing and onconeuronal and cell surface antibodies analysis, which were also performed in serum, were negative. An electrophysiological evaluation performed 24 hours after the initial neurological examination suggested an acute inflammatory demyelinating polyneuropathy, following the Uncini criteria of GBS [3], with an increase in distal motor latency and conduction block in both median nerves, absent F-response for peroneal and tibial nerves, and absence of median and ulnar nerve sensory nerve action potential with normal sensory conductions in lower-extremities. It also showed a severe drop in the amplitude of bilateral facial nerve motor potentials. Normality in repetitive nerve stimulations at low and high frequencies, and single-fiber electromyography (EMG), were observed. Concurrently, an extensive systemic infection screening yielded negative results. Considering these results, vascular, infectious, tumoral and paraneoplastic brainstem encephalitis (Table 1) were reasonably excluded.

**Table 1.** **Differential diagnosis of multiple cranial affectation with cerebellar syndrome**.

Brainstem injury	
Etiology	Complementary tests
Vascular	-CT
∙ Ischemic	-MRI
∙ Hemorrhagic	-CSF analysis
Neoplastic	-LDH, ß2 microglobulin
∙ Glioma	
∙ Lymphoma	
∙ Metastases	
Infection/Rhombencephalitis*	-Blood and CSF analysis
∙ Viruses: Herpes zoster, Epstein-Barr, Cytomegalovirus, HIV 1&2	-Stool analysis
∙ Bacterial: Listeria Monocytogenes, Treponema pallidum, Lyme	
∙ Other: Pseudomonas sp, Mycobacterium tuberculosis, fungal, parasites	
Inflammatory-autoimmune*	-Antibodies determination: Anti-aquaporin, anti-MOG, anti-neuronal (i.e., Anti-Hu, anti-Ri) and cell surface (i.e., anti-NMDA, anti-IgLON5), antinuclear and anti-DNA, antiganglioside.
∙ Paraneoplastic syndromes	
∙ Systemic disease: Behçet’s disease, systemic lupus erythematosus	
∙ Demyelinating: Multiple sclerosis, NMOSD/MOGAD, CLIPPERS	
∙ Bickerstaff encephalitis	-Electrophysiological study
Toxic-metabolic	-Clinical diagnosis
∙ Wernicke encephalopathy	
Multiple cranial neuropathy	
Etiology	Complementary tests
Tumoral	-CT
∙ Carcinomatous meningitis	-MRI
	-CSF analysis
Infectious*	-Gram stain, culture, toxin testing
∙ Diphtheria	-Chest and neck X-ray
Inflammatory-autoimmune*	-MRI
∙ Vasculitis	-PET-scan
∙ Systemic: sarcoidosis	-Systemic focused-evaluation (cardiac, ocular, thoracic)
∙ Fisher-syndrome	-Angiotensin-converting enzyme, liver biochemistry
	-Electrophysiological study
Neuromuscular junction disorders	
Etiology	Complementary tests
Botulism	-Toxin determination
	-Electrophysiological study
Miastenia Gravis	-Antibodies determination: AChR-Ab, MuSK-Ab, LRP4-Ab
	-Electrophysiological study
	-Thoracic CT

Depending on the lesion topography, different etiologies should be suspected. Etiologies indicated with (*) can manifest as a brainstem injuries or multiple cranial neuropathies, constituting a spectrum in which some tend to present as the first and others as the second one. *Abbreviations:* CT, Computed Tomography; MRI, Magnetic Resonance Imaging; CSF, Cerebrospinal Fluid; HIV, Human Immunodeficiency Virus; NMOSD, neuromyelitis optica spectrum disorders; MOGAD, myelin oligodendrocyte glycoprotein antibody-associated disease; CLIPPERS, chronic lymphocytic inflammation with pontine perivascular enhancement responsive to steroids; LDH Lactate dehydrogenase; PET-scan, Positron Emission Tomography-scan; AChR-Ab, Acetylcolinesterase Receptor antibody; MuSK-Ab, Muscle-Specific Kinase antibody; LRP4-Ab, Low density lipoprotein Receptor-related Protein 4 antibody; MOGAD, myelin oligodendrocyte glycoprotein antibody-associated disease; NMDA, Anti-N-methyl-D-aspartate.

**Fig. 1.**
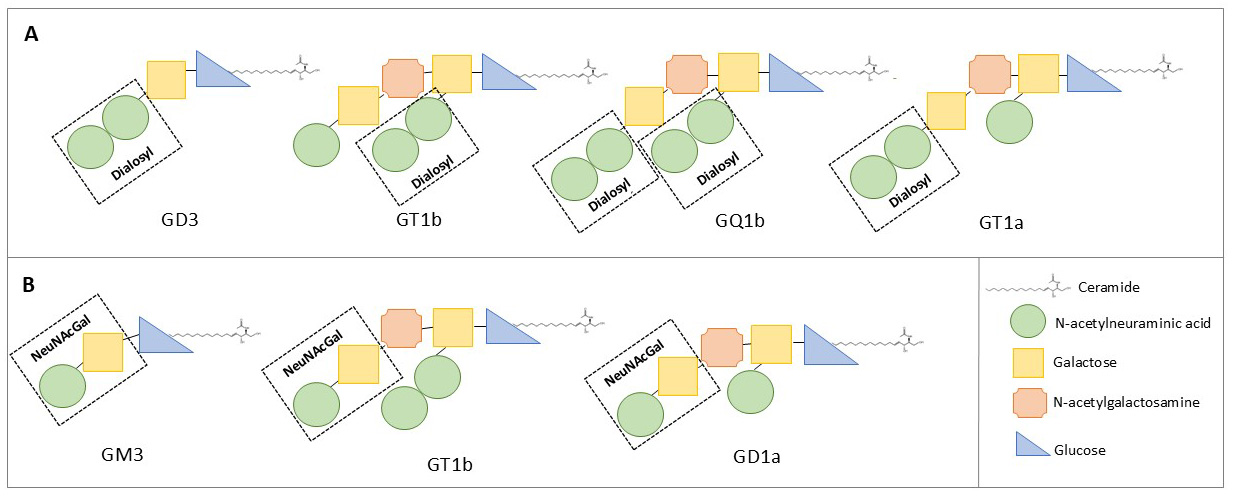
**Structure of the different gangliosides with antibody reactivity in our patient**. In (A) is represented the dialosyl group; in (B) the terminal NeuNAc(α2-3)Gal. Adapted from [4, 5].

The onset of neurological symptoms, the previous infectious disorder, and the Nerve Conduction Studies (NCS) led to consideration of a dysimmune disorder within the Fisher-spectrum. Therefore, treatment with intravenous immunoglobulins for 5 days was initiated and antiganglioside antibodies were obtained. Results showed reactivity against multiple gangliosides, with antibodies positivity against two different epitopes (Fig. 1): the disialosyl group (anti-trisialosylganglioside GT1a>1/12500; anti-trisialosylgangliosid GT1b>12500; anti-tetrasialosylgangliosid GQ1b>12500; antidialosylgangliosid GD3>1/12500), and the terminal NeuNAc(α2-3)Gal (IgG anti-dialosylgangliosid GD1a 1/2118; anti-trisialosylgangliosid GT1b>12500, anti-monosialosylgangliosid GM3>1/2500) [4, 5].

A progressive improvement was observed. At 5 months, sequelae included asymmetric non-reactive mydriasis, bilateral ocular esotropia with diplopia on left and upper gaze, mild tongue atrophy, and moderate dysarthria. Also, NCS showed a recovery of the previously altered findings.

## 3. Discussion

This case underscores the remarkable phenotypic variability of GBS. The patient’s presentation, featuring a combination of ophthalmoplegia, ataxia, facial diplegia, and acute bulbar palsy, is highly atypical. It emphasizes the importance of conducting a thorough differential diagnosis to distinguish it from other neurological disorders that may present similarly.

As previously described in literature [6, 7], there appears to be a correlation between GBS clinical phenotype and antiganglioside antibody specificity, likely depending on the distribution of ganglioside antigens in the peripheral nervous system, which can be observed in this case report.

The presence of anti- GQ1b, detected in ≥90% of Miller Fisher patients’ serum [8], may elucidate the complete ophthalmoplegia and ataxia in the patient. This can be attributed to its high expression at the paranodes and neuromuscular junctions of the oculomotor, trochlear and abducens nerves, as well as in the group Ia afferents in muscle spindles.

Reactivity against NeuNAc(α2-3)Gal is rare (<0.3%), typically reported along with concurrent positivity for terminal dialosyl, making isolated reactivity exceptionally uncommon (<0.03%) [9]. Only 1 out of 10 cases in the literature [10] exhibited an isolated reactivity against NeuNAc(α2-3)Gal gangliosides. In the remaining 9 out of 10 cases, sera reacted with other gangliosides, showing dual reactivity against dialosyl and NeuNAc (α2-3)Gal in 8/9 instances. Reactivity versus NeuNAc(α2-3)Gal was frequently associated with bulbar symptoms, presenting acutely with IgG antibodies (5/8), similar to those reported in the patient [9]. Concerning anti-GT1a, it is also linked with cranial nerve abnormality (n = 17/23; *p* = 0.001), with oropharyngeal weakness being the most prominent sign (n = 16/23; *p* < 0.001) [8]. Therefore, acute bulbar palsy could be explained by antibodies targeting NeuNAc(α2-3) Gal and GT1a.

Lastly, evidence-based on clinical trials support immunotherapy for GBS, with intravenous immunoglobulin and plasma exchange being proven therapies equally efficacious in its management [11, 12]. One or the other should be initiated as soon as GBS is considered, even in the absence of electrophysiological studies or antibody test results.

## 4. Conclusions

In conclusion, our case illustrates reactivity to multiple gangliosides, highlighting the shared epitopes among these molecules and the ability of a single antibody to target various types of gangliosides, while also underscoring an extremely rare GBS phenotype.

## Data Availability

Anonymized data from this study will be shared at the request of any qualified investigator.
