# Supplementary material for: A Rare Guillain-Barré Syndrome Variant with Multi-Ganglioside Reactivity: A Case of Severe Cranial Nerve Involvement
Source: Rev Neurol. 2025 Feb 12;80(1):37744. doi: 10.31083/RN37744 (PMC11907704; doi:10.31083/RN37744)
Supplement: Translation

# Translation: Variabilidad Fenotípica y Especificidad de los Anticuerpos en el Síndrome de Guillain-Barré: Reporte de un Caso

**Published:** 2025-02-12

**Authors:** Laura Gómez-Dabó, Arnau Llaurado, Daniel Sánchez-Tejerina, Victoria González, Carmen Montalvo-Olmedo, Carlos Lázaro-Hernández, Marc Rodrigo-Gisbert, Samuel López-Maza, Maider Iza-Achutegui, Lídia Giramé-Rizzo, Nuria Raguer, Raúl Juntas

**Affiliations:** ^4^Departamento de Neurología, Hospital Universitario Vall d’Hebron, Universitat Autònoma de Barcelona, 08035 Barcelona, ​​España; ^5^Unidad de Enfermedades Neuromusculares, Servicio de Neurología, Hospital Universitario Vall d’Hebron, 08035 Barcelona, ​​España; ^6^Departamento de Neurofisiología Clínica, Hospital Universitario Vall d’Hebron, 08035 Barcelona, ​​España

**Keywords:** anticuerpo anti-gangliósido, disialosilo, síndrome de Guillain-Barré, multineuritis, NeuNAc(α2-3)Gal, neuropatía

## Abstract

**Introducción:**

Presentamos un caso clínico de polirradiculoneuritis aguda inmunomediada que inicialmente se manifestó con oftalmoparesia-ataxia, diplegia facial y parálisis bulbar aguda, acompañada de un perfil autoinmune característico.

**Caso Clínico:**

Describimos el caso de una mujer de 75 años con clínica de progresión rápida incluyendo midriasis bilateral no reactiva, ptosis, oftalmoplejía completa, paresia facial bilateral, paresia lingual, parálisis del paladar, dismetría en todas las extremidades, ataxia y reflejos osteotendinosos aumentados de forma generalizada, con nivel de conciencia preservado. La clínica inició después de una posible infección gastrointestinal de aparición diez días antes. Su estado clínico empeoró rápidamente, requiriendo intubación orotraqueal y traqueotomía debido a un compromiso bulbar severo. La afectación concomitante de múltiples nervios craneales sugirió una lesión en el tronco encefálico, destacándose la parálisis oculomotora y bulbar aguda. La resonancia magnética del tronco encefálico y médula espinal, junto con las pruebas de líquido cefalorraquídeo, no mostraron alteraciones; la electromiografía objetivó una polirradiculoneuropatía desmielinizante sensitivo-motora. La prueba de anticuerpos antigangliósidos mostró positividad contra múltiples anticuerpos dirigidos al grupo dialosilo y al terminal NeuNAc(α2-3)Gal. El tratamiento con inmunoglobulinas se asoció a una mejoría gradual.

**Conclusiones:**

Nuestro caso ilustra la reactividad a múltiples gangliósidos, destacando los epítopos compartidos entre estas moléculas y la capacidad de un único anticuerpo para dirigirse a diversos tipos de gangliósidos, subrayando además un fenotipo extremadamente raro del síndrome de Guillain-Barré.

## 1. Introducción

El síndrome de Guillain-Barré (SGB) es un trastorno clínicamente heterogéneo caracterizado por una presentación clásica en forma de debilidad progresiva ascendente en las extremidades con reflejos disminuidos o ausentes. Si bien los criterios diagnósticos actuales [1, 2] permiten realizar el diagnóstico de la mayoría de los pacientes con SGB, una proporción significativa no cumple con estos criterios. En tales casos, los estudios neurofisiológicos completos y las pruebas de anticuerpos anti-gangliósidos pueden resultar especialmente útiles.

En este caso clínico, presentamos a una paciente con polirradiculoneuritis aguda inmunomediada, que debutó con oftalmoparesia-ataxia, diplegia facial y parálisis bulbar aguda, acompañada de un perfil autoinmune característico.

## 2. Caso Clínico

Una mujer de 75 años con antecedentes de hipertensión, dislipidemia y asma ingresó en el Departamento de Medicina Interna debido a una probable infección gastrointestinal. No se identificó ningún patógeno específico, aunque se inició tratamiento empírico con amoxicilina-clavulánico, resultando en la remisión de los síntomas y la fiebre en cinco días. Sin embargo, dos días después, presentó un rápido deterioro clínico con afectación bilateral de los pares craneales III, IV, VI, VII, IX, X y XII. Clínicamente se manifestó en forma de oftalmoplejía bilateral con ptosis completa, midriasis no reactiva, parálisis facial, parálisis lingual, ausencia de elevación del paladar, reflejo faríngeo abolido y disartria flácida. El examen también reveló reflejos osteotendinosos aumentados y dismetría en miembros superiores e inferiores. No se objetivaron alteraciones en el examen en cuanto al balance motor, la sensibilidad ni el nivel de conciencia. En pocas horas, la paciente empeoró con insuficiencia respiratoria, requiriendo intubación endotraqueal e ingreso en la unidad de cuidados intensivos.

La Resonancia Magnética (RM) urgente cerebral y de la médula espinal objetivaron cambios crónicos leves e inespecíficos en la sustancia blanca cerebral, con parénquima normal del tronco encefálico y médula espinal, sin realce de gadolinio y con permeabilidad vascular en la angiografía por resonancia. La punción lumbar con estudios de citología y bioquímica del líquido cefalorraquídeo (LCR) resultó normal (0 células/uL, glucosa 86 mg/dL, proteína 38 mg/dL). Se realizaron pruebas microbiológicas extensas del LCR y análisis de anticuerpos onconeuronales y de superficie celular, también realizados en suero, que fueron negativos. Una evaluación electrofisiológica realizada 24 horas después del examen neurológico inicial sugirió una polineuropatía inflamatoria desmielinizante aguda, siguiendo los criterios de Uncini para SGB [3], con un aumento en la latencia motora distal y bloqueo de conducción en ambos nervios medianos, ausencia de respuesta F para los nervios peroneo y tibial, y ausencia de potencial de acción sensorial en los nervios mediano y cubital, con conducciones sensoriales normales en las extremidades inferiores. También mostró una disminución severa en la amplitud de los potenciales motores del nervio facial bilateral. La estimulación repetitiva del nervio y el electromiografía (EMG) de fibra única fueron normales. Paralelamente, se realizó una evaluación exhaustiva de infección sistémica que resultó negativa. Con todo ello, se excluyó razonablemente una encefalitis tronco-encefálica de causa vascular, infecciosa, tumoral y paraneoplásica (Tabla 1).

**Tabla 1.** **Diagnóstico diferencial de la afectación de múltiples pares craneales asociada con síndrome cerebeloso**.

Encefalitis troncoencefálica	
Etiología	Pruebas complementarias
Vascular	-TC, RM
∙ Isquémica	-Análisis LCR
∙ Hemorrágica	-LDH, ß2-microglobulina
Neoplásica	
∙ Glioma	
∙ Linfoma	
∙ Metástasis	
Infección/Romboencefalitis*	-Análisis de suuero y LCR
∙ Virus: Herpes zoster, Epstein-Barr, Citomegalovirus, VIH 1 y 2	-Coprocultivo
∙ Bacterias: *Listeria monocytogenes, Treponema pallidum,* enfermedad de Lyme	
∙ Otros: Pseudomonas sp, *Mycobacterium tuberculosis*, hongos, parásitos	
Inflamatoria-autoinmune*	-Determinación de anticuerpos: Anti-aquaporina, anti-MOG, anti-neuronales (ej. Anti-Hu, anti-Ri) y anti-superfície (ej. anti-NMDA, anti-IgLON5), anti-nucleares y anti-DNA, anti-gangliósidos.
∙ Síndromes paraneoplásicos	
∙ Enfermedades sistémicas: enfermedad Behçet, lupus eritematoso sistémico	
∙ Desmielinizantes: esclerosis múltiple, NMOSD/MOGAD, CLIPPERS	
∙ Encefalitis de Bickerstaff	-Estudio electrofisiológico
Tóxico-metabólica	-Diagnóstico clínico
∙ Encefalopatía de Wernike	
Neuropatía múltiple craneal	
Etiología	Pruebas complementarias
Tumoral	-TC, RM
∙ Carcinomatosis meníngea	-Análisis LCR
Infecciosa*	-Tinción de Gram, cultivo, determinación de la toxina
∙ Difteria	-Rayos X incluyendo tórax y cuello
Inflamatoria-autoinmune*	-RM
∙ Vasculitis	-PET-scan
∙ Sistémica: sarcoidosis	-Evaluación sistémica (cardíaca, ocular, torácica)
∙ Espectro Fisher	-Enzima convertidora angiotensina, enzimas hepáticas, bioquímica
	-Estudio neurofisiológico
Trastornos de la unión neuromuscular	
Etiología	Pruebas complementarias
Botulismo	-Determinación toxina
	-Estudio electrofisiológico
Miastenia Gravis	-Determinación de anticuerpos: anti-AChR, anti-MuSK, anti-LRP4
	-Estudio electrofisiológico
	-TC torácico

Dependiendo de la topografía de la lesión, diferentes etiologías deberían sospecharse. Las etiologías señaladas con (*) se pueden manifestar como encefalitis troncoencefálica o como neuropatía craneal múltiple, constituyendo un espectro en que algunos se manifiestan de la primera y otros de la segunda forma. Abreviaciones: anti-AChR, anticuerpo anti-receptor de acetilcolina; anti-LRP4, anticuerpo anti receptor de lipoproteína de baja densidad; anti-MOG, anticuerpo contra el receptor de NMDA; anti-MusK, anticuerpo anti-tirosinfosfokinasa; CLIPPERS, *chronic lymphocytic inflammation with pontine perivascular enhancement responsive to steroids; *LDH, Lactato Deshidrogenasa; LCR, Líquido Cefalorraquídeo; MOGAD, enfermedad por anticuerpos MOG; NMOSD, Trastorno de Espectro Neuromielitis Óptica; PET-scan, Tomografía de Emisión de Positrones; RM, Resonancia Magnética; TC, Tomografía Computerizada; VIH, Virus de Inmunodeficiencia Humana.

El inicio de los síntomas neurológicos precedidos de una probable infección gastrointestinal, junto con los estudios de conducción nerviosa, sugirieron un trastorno disinmune dentro del espectro Fisher. Se inició tratamiento con inmunoglobulinas intravenosas durante 5 días y se obtuvieron anticuerpos anti-gangliósidos. Los resultados mostraron reactividad contra múltiples gangliósidos, con positividad de anticuerpos contra dos epítopos diferentes (Fig. 1): el grupo disialosilo (anti-trisialosilgangliósido GT1a>1/12500; anti-trisialosilgangliósido GT1b>12500; anti-tetrasialosilgangliósido GQ1b>12500; anti-dialosilgangliósido GD3>1/12500) y el terminal NeuNAc(α2-3)Gal (IgG anti-dialosilgangliósido GD1a 1/2118; anti-trisialosilgangliósido GT1b>12500, anti-monosialosilgangliósido GM3>1/2500) [4, 5].

**Fig. 1.** **Estructura de los diferentes gangliósidos y reactividad de los anticuerpos anti-gangliósido del caso**. In (A) se representa el grupo dialosil; en (B) el terminal NeuNAc(α2-3)Gal. Adaptado de [4, 5].

Tras el inicio del tratamiento, se observó una mejoría progresiva. A los 5 meses, las secuelas incluían midriasis asimétrica no reactiva, esotropía ocular bilateral con diplopía en levoversión y supraversión, atrofia lingual leve y disartria moderada. Paralelamente, los estudios de conducción nerviosa mostraron una recuperación de los hallazgos previamente alterados.

## 3. Discusión

Este caso destaca la notable variabilidad fenotípica del SGB. El debut clínico de la paciente con oftalmoplejía, ataxia, diplegia facial y parálisis bulbar aguda es altamente atípico. El caso subraya la importancia de realizar un diagnóstico diferencial exhaustivo para distinguirlo de otros trastornos neurológicos que pueden presentar síntomas similares.

Como se ha descrito en la literatura [6, 7], parece existir una correlación entre el fenotipo clínico del SGB y la especificidad de los anticuerpos anti-gangliósidos, probablemente secundario a la distribución de los antígenos de gangliósidos en el sistema nervioso periférico, como se observa en este caso.

La positividad ante anti-GQ1b, detectado en ≥90% del suero de pacientes con el síndrome de Miller Fisher [8], podría explicar la oftalmoplejía completa y la ataxia en la paciente. Esto puede atribuirse a su alta expresión a nivel paranodal y en las uniones neuromusculares de los pares craneales oculomotor, troclear y abducens, así como en los aferentes del grupo Ia en los husos musculares.

La reactividad contra NeuNAc(α2-3)Gal es rara (<0,3%), y típicamente se reporta junto con una positividad concurrente para el terminal dialosil, siendo la reactividad aislada excepcionalmente rara (<0,03%) [9]. Solo 1 de cada 10 casos en la literatura [10] ha presentado una reactividad aislada contra los gangliósidos NeuNAc(α2-3)Gal. La reactividad frente a NeuNAc(α2-3)Gal se asoció frecuentemente con síntomas bulbares, presentándose de manera aguda con anticuerpos IgG (5/8), similar a los observados en la paciente [9]. En cuanto al anti-GT1a, también se ha asociado con anomalías en los pares craneales (n = 17/23; *p* = 0.001), siendo la debilidad orofaríngea el signo más prominente (n = 16/23; *p* < 0.001) [8]. Por lo tanto, la parálisis bulbar aguda podría explicarse por anticuerpos dirigidos contra NeuNAc(α2-3)Gal y GT1a.

Por último, la evidencia basada en ensayos clínicos respalda la inmunoterapia para el SGB, siendo la inmunoglobulina intravenosa y la plasmaféresis terapias comprobadas y de igual eficacia en su manejo [11, 12]. Una u otra debería iniciarse tan pronto como se considere la posibilidad de SGB, incluso en ausencia de estudios electrofisiológicos o resultados de pruebas de anticuerpos.

## 4. Conclusiónes

En conclusión, nuestro caso enfatiza en la posibilidad de presentar reactividad a múltiples gangliósidos, destacando los epítopos compartidos entre estas moléculas y la capacidad de un solo anticuerpo para dirigirse a diversos tipos de gangliósidos, al tiempo que describe un fenotipo extremadamente raro del SGB.

## Data Availability

Anonymized data from this study will be shared at the request of any qualified investigator.

## Abbreviations

CSF, cerebrospinal fluid; EMG, electromyography; GBS, Guillain-Barré Syndrome; MRI, Magnetic Resonance Imaging; NCS, Nerve Conduction Studies.

## Supplementary Material

Supplementary material associated with this article can be found, in the online version, at https://doi.org/10.31083/RN37744.

## References

[1] Shahrizaila N, Lehmann HC, Kuwabara S (2021). Guillain-Barré syndrome. *Lancet (London, England)*.

[2] Fokke C, van den Berg B, Drenthen J, Walgaard C, van Doorn PA, Jacobs BC (2014). Diagnosis of Guillain-Barré syndrome and validation of Brighton criteria. *Brain: a Journal of Neurology*.

[3] Uncini A, Ippoliti L, Shahrizaila N, Sekiguchi Y, Kuwabara S (2017). Optimizing the electrodiagnostic accuracy in Guillain-Barré syndrome subtypes: Criteria sets and sparse linear discriminant analysis. *Clinical Neurophysiology: Official Journal of the International Federation of Clinical Neurophysiology*.

[4] Shahrizaila N, Yuki N (2013). Bickerstaff brainstem encephalitis and Fisher syndrome: anti-GQ1b antibody syndrome. *Journal Of Neurology Neurosurgery And Psychiatry*.

[5] Willison HJ, Yuki N (2002). Peripheral neuropathies and anti-glycolipid antibodies. *Brain*.

[6] Lleixà C, Martín-Aguilar L, Pascual-Goñi E, Franco T, Caballero M, de Luna N (2021). Autoantibody screening in Guillain-Barré syndrome. *Journal of Neuroinflammation*.

[7] Goodfellow JA, Willison HJ (2016). Antiganglioside, antiganglioside-complex, and antiglycolipid-complex antibodies in immune-mediated neuropathies. *Current Opinion in Neurology*.

[8] Kim JK, Bae JS, Kim DS, Kusunoki S, Kim JE, Kim JS (2014). Prevalence of anti-ganglioside antibodies and their clinical correlates with guillain-barré syndrome in Korea: a nationwide multicenter study. *Journal of Clinical Neurology (Seoul, Korea)*.

[9] Rojas-Garcia R, Gallardo E, De Luna N, Juarez C, Martinez-Hernandez E, Carvajal A (2010). Bulbar involvement in patients with antiganglioside antibodies against NeuNAc(alpha2-3)Gal. *Journal of Neurology, Neurosurgery, and Psychiatry*.

[10] Rojas-Garcia R, Martinez-Lage M, Gallardo E, de Luna N, Marti-Vilalta JL, Diaz-Manera J (2006). A novel antiganglioside specificity against terminal NeuNAc(alfa 2-3)Gal in acute bulbar palsy. *Journal of Neuroimmunology*.

[11] van der Meché FG, Schmitz PI (1992). A randomized trial comparing intravenous immune globulin and plasma exchange in Guillain-Barré syndrome. Dutch Guillain-Barré Study Group. *The New England Journal of Medicine*.

[12] McKhann GM, Griffin JW, Cornblath DR, Mellits ED, Fisher RS, Quaskey SA (1988). Plasmapheresis and Guillain-Barré syndrome: analysis of prognostic factors and the effect of plasmapheresis. *Annals of Neurology*.
